# A Monocular Variable Magnifications 3D Laparoscope System Using Double Liquid Lenses

**DOI:** 10.1109/JTEHM.2023.3311022

**Published:** 2023-09-01

**Authors:** Fan Mao, Tianqi Huang, Longfei Ma, Xinran Zhang, Hongen Liao

**Affiliations:** Department of Biomedical EngineeringSchool of MedicineTsinghua University 12442 Beijing 100084 China

**Keywords:** Deep learning network, depth from defocus (DFD), laparoscope, minimal invasive surgery (MIS), 3D reconstruction

## Abstract

During minimal invasive surgery (MIS), the laparoscope only provides a single viewpoint to the surgeon, leaving a lack of 3D perception. Many works have been proposed to obtain depth and 3D reconstruction by designing a new optical structure or by depending on the camera pose and image sequences. Most of these works modify the structure of the conventional laparoscopes and cannot provide 3D reconstruction of different magnification views. In this study, we propose a laparoscopic system based on double liquid lenses, which provide doctors with variable magnification rates, near observation, and real-time monocular 3D reconstruction. Our system composes of an optical structure that can obtain auto magnification change and autofocus without any physically moving element, and a deep learning network based on the Depth from Defocus (DFD) method, trained to suit inconsistent camera intrinsic situations and estimate depth from images of different focal lengths. The optical structure is portable and can be mounted on conventional laparoscopes. The depth estimation network estimates depth in real-time from monocular images of different focal lengths and magnification rates. Experiments show that our system provides a 0.68-1.44x zoom rate and can estimate depth from different magnification rates at 6fps. Monocular 3D reconstruction reaches at least 6mm accuracy. The system also provides a clear view even under 1mm close working distance. Ex-vivo experiments and implementation on clinical images prove that our system provides doctors with a magnified clear view of the lesion, as well as quick monocular depth perception during laparoscopy, which help surgeons get better detection and size diagnosis of the abdomen during laparoscope surgeries.

## I. Introduction

Laparoscopes are used in minimally invasive surgeries, can help doctors diagnose diseases in the abdomen or pelvis, and perform surgical procedures, including removing damaged or diseased organs or taking tissue samples for further testing. Obtaining different fields of view and having depth perception during laparoscopic surgeries are critical. Conventional laparoscopes are designed with a fixed magnification rate and are moved forward and backward by the assistant to obtain a magnified vision of the organ, which makes it tiring during long surgery hours and may cause a collision of instruments. Although many current techniques propose designing stereo structures, structure light devices, or using deep learning methods to conduct 3D reconstruction of the laparoscopic scenes, these methods depend greatly on the device itself and do not provide multi-magnifications for 3D reconstruction. In addition, ways that provide multi-magnification in laparoscopic surgeries mainly customize the optical structure of the laparoscope to provide a wider field of view, and therefore they cannot be applied to all conventional laparoscopes. To obtain automated variable magnifications and corresponding monocular 3D reconstruction applicable to all conventional laparoscopes, designing a portable optical device with automated algorithms is a solution.

3D reconstruction in laparoscopic surgeries is very critical in minimal invasive surgeries (MIS). Works have shown that real time 3D visualization and reconstruction reduces operation time, compared to high-definition 2D in laparoscopic liver resection [1]. 3D imaging improves the accuracy of dissection and better intra-corporeal knotting. It is also proven to provide better clinical outcomes in urology [2], laparoscopic right colectomy [3], and laparoscopic gastrectomy for gastric cancer [4]. The 3D reconstruction of a wide view helps localize surgical instruments and the one of a magnified view provides doctors with 3D morphology recognition of organs and neoplasms. Many 3D reconstruction methods have been proposed for laparoscopes, mainly classified into active and passive methods [5]. Kim et al. [6] designed a two-camera system that provides a panoramic view and depth perception using stereo cues. Sui et al. [7] further proposed a stereo structure with structure light for 3D reconstruction. These active methods depend greatly on the stereo system [8] and the projected patterns [9]. Furthermore, stereo and structured light methods have working distance limitations and fail to provide close observations, which are critical in fluorescence imaging, especially in colonoscopy [10].

On the other hand, passive methods depend mainly on the images acquired. Many passive laparoscopic 3D reconstruction methods are similar to those used in natural images, Collins and Bartoli [11] used shape from shading to estimate 3D shapes in real-time from monocular laparoscopy videos. Nakajima et al. [12] used shape from focus to conduct 3D shape measurement in monocular endoscopy. Widya et al. [13] used structure from motion to reconstruct gastric organs. Lamarca et al. [14] proposed using Simultaneous Localization and Mapping (SLAM) to reconstruct deformable monocular sequences. Recent work on passive methods mainly focuses on deep learning means. Shao et al. [15] and Liu et al. [16] both designed a self-supervised deep-learning network for depth and ego-motion estimation. They also added appearance flow for better correspondence. Bardozzo et al. [17] introduced a self-supervised network that can conduct both mono and stereo real-time depth estimation. Liu et al. [18] proposed a self-supervised network that learns depth from monocular endoscopy videos through features. The disadvantages of the above passive methods are that they depend greatly on camera intrinsic and feature cues, which are difficult to obtain in real surgical scenes. The reconstructed scenes are based on a fixed field of view, providing the same reconstruction resolution between different reconstruction frames and simply overlayed and stitched.

To provide multi-magnifications and near observation which help give a clearer observation of lesions and neoplasms, various optical systems have been designed to widen the field of view and provide autofocus. However, these systems are rather bulky. As conventional laparoscopes offer a fixed field of view and depth of view, their working distance does not guarantee close observations under 3mm. Assistants adjust the focus of the laparoscope before surgeries by rotating the ring on the imaging lens group to ensure clear imaging. To provide a more automated way of focusing, Liu et al. [18] applied minimized motors to conduct quick autofocus. However, this method still needs the physical movement of the optical lenses. Zou et al. [19] designed Alvarez lenses that can conduct focus range without physical movements, but the micromachining technique of the lenses is complex. Wang et al. [20] demonstrated a 3D integral-imaging endoscopy with tunable DOF by using a single large-aperture focal-length-tunable liquid crystal (LC) lens. Kanhere et al. [21] introduced a multi-camera system to capture different views and stitch them together for a wider view. The above-mentioned methods add complexity to the laparoscope. To obtain automated focusing and provide a wider view range, a liquid lens-based design is also proposed as a more practical alternative. Volpi et al. [22] designed an optical structure containing a liquid lens that provides clear observation of fluorescent imaging under near working distances.

To further provide variable magnification rates, a combination of multiple liquid lenses is proposed. Qin et al. [23] proposed an optical structure with two liquid lenses which can obtain a magnification range from 2x to 3x. Li et al. [24] also proposed using two liquid lenses, but with fewer optical lenses, and can also obtain 3x magnification. However, the above structures are long, add weight to the conventional laparoscope, and may require customized lenses. Magnified views also suffer from poor light, chromatic and spherical aberration, as well as astigmatism. Additional optical optimizations are required. Katz et al. [25] conducted optimization of the double-liquid lens structure Qin proposed and reduced chromatic aberration. He also dealt with the poor light in the high magnification rate of the optical system [26]. The more optical elements the system contains, the more it is vulnerable to light conditions and the more optimization it should need.

Considering the drawbacks of the aforementioned methods, namely the inability to fit conventional laparoscopes, and the dependency on camera intrinsic and the surgical environment, a new portable and light-weighted optical structure using off-the-shelf lenses which can be easily mounted between conventional laparoscopes and the camera is proposed. A depth estimation network based on the depth from defocus technique is also introduced to laparoscopy field to fit the multi-magnifications system.

In this paper, we design a laparoscopic system that provides multi-magnification, auto-focus, near observation, and depth estimation. We summarize our contributions as follows:

• A novel optical structure with two liquid lenses and three optical lenses is proposed that enables direct integration into the rear of a conventional laparoscopic lens group and provides multi-magnification, autofocus and close-up viewing for laparoscopes. The structure uses off-the-shelf lenses and is lightweight. The entire optical structure is tailored to the specific working distance and field of view of conventional laparoscopes. This customization ensures seamless integration into laparoscopic procedures.
• Our algorithm innovatively addresses the challenge of varying camera intrinsic properties with changes in magnification in endoscopic depth estimation, achieved through the introduction of a real-time depth-from-defocus (DFD) network. A specialized preprocessing method is introduced to simulate laparoscopic blur conditions, enhancing accuracy. Notably, our work enables real-time laparoscopic depth estimation using a DFD network for the first time.

## II. Methods and Procedures

**FIGURE 1.**
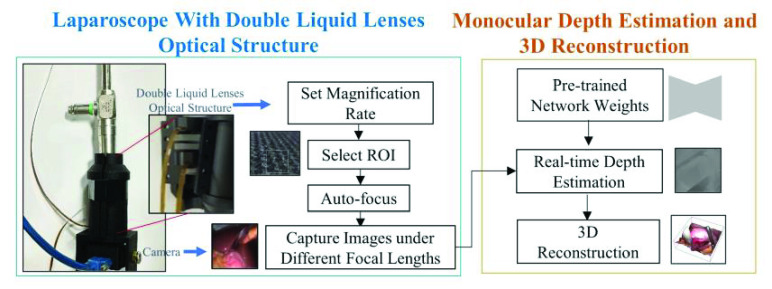
The proposed methods and framework of the monocular variable magnifications 3D laparoscope system include two parts: the double liquid lenses optical structure (blue), mounted on the laparoscope, and the monocular depth estimation network (yellow). The magnification rate and ROI is selected on the computer and control commands are sent to the double liquid lenses to conduct quick auto-focus and capture monocular images. The captured monocular images under different focal lengths are then inputted into the monocular depth estimation network, which outputs the corresponding depth and conduct 3D reconstruction.

To realize both multi-magnification and 3D reconstruction in one system, an optical structure and a network are proposed. The overview of our system is shown in Fig. 1. Our system integrates a double-liquid-lenses optical structure and a monocular depth estimation network. An optical structure using two liquid lenses is designed, which provide close observations suitable for fluorescence scenes, multi-magnification range, and auto-focusing. The optical structure’s imaging effect, including chromatic aberration, astigmatism, and comet influence, is optimized.

A monocular depth estimation network trained using the DFD technique is introduced to laparoscopic scenes to cope with inconsistent camera intrinsic situations. It can estimate depth from the images captured by the optical structure, which are of different focal lengths and magnification rates.

### A. Double Liquid Lenses Optical System Design

**FIGURE 2.**
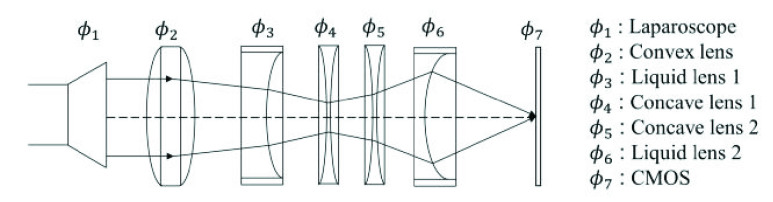
Optical design of double liquid lens system.

We develop an innovative optical configuration comprising two liquid lenses and three auxiliary lenses positioned between the conventional laparoscopic assembly and the CMOS chip. This arrangement seamlessly integrates into the traditional laparoscopic system, effectively enabling versatile functionalities including multi-magnification, automated focusing, and close-range observation. The optical configuration is meticulously customized to harmonize with the working distance and field of view intrinsic to conventional laparoscopes. Consequently, we propose an optical configuration composed of five distinct lenses, comprising two liquid lenses, one convex lens, and two concave lenses, as depicted in Fig. 2.

The conventional laparoscope used is a 0°, 325 mm long, 10mm diameter, and 70° field of view laparoscope (Shenda J0800B SN 350.0110). A 1/1.8” charge-coupled device (CCD) is used. The required focal length of the system can be calculated according to the following Equation (1), in which $f$ is the focal length of the system, *WD* is the working distance of the system, *FOV* is the field of view of the system, and *CCD* is the size of the camera.

\begin{equation*} f=\frac {WD}{\frac {FOV}{CCD}+1} \tag{1}\end{equation*}

Based on the focal length requirement above, an optical configuration composed of five distinct lenses, comprising two liquid lenses, one convex lens, and two concave lenses is proposed to accomplish the magnification adjustment without physical movements.

The first lens should add adequate optical power to the optical structure and help minimize both chromatic aberration and spherical aberration. Results show that placing a convex lens as the first lens has less effect on speeding the drop of the MTF curve. After several experiments, a doublet with a focal length of 60mm is chosen. It is then followed by an Optotune EL-10-30-TC liquid lens. As the first tunable lens, it works as a focusing lens, which provides a diopter range from 8 dpt to 20 dpt. The third and fourth lenses should help diffuse the light rays so that they can intersect on the image plane. Two biconcave lenses are chosen as they show a better effect on reducing astigmatism than two plano-concave lenses. The combination of two lenses with the same refractive index can also help minimize chromatic aberration. After several experiments, the focal length of −180mm each is set.

The two biconcave lenses are then followed by another Optotune EL-10-30-TC liquid lens, which acts as a magnification ranging lens, by the back-lens configuration requirement.

The structure is accommodated to the working distance and field of view of conventional laparoscopes, the overall structure is then optimized using Zemax optical ray-tracing software. After deciding the focal lengths and the sequences of the lenses in our system, the distances between the lenses are set as variables and the optimizing function in Zemax is further used to optimize these distances to achieve a better image. The weight of the focal length (EFFL), spherical aberration (SPFA), coma (COMA), and astigmatism (ASTI) are set at 1.0 and these constraints are used as our merit function optimization. As the lenses are all off the shelf, the thickness and radius of our system cannot be further optimized. The merit function of our system dropped to 0.969 after 10 cycles. The imaging resolution and contrast of the system have been optimized. After optimization, the influence of chromatic aberration, astigmatism, and comet on the optimized system is greatly reduced. The overall focal length of the system is 46mm-86mm, and provides clear view under 1mm-200mm working distance.

Python is used to control the focal length of the liquid lens. This entails the transmission of specific commands directed at modulating the electric current traversing the liquid lens, consequently orchestrating a corresponding adjustment in its focal length. Notably, this intricate process is executed within an impressively expedited interval of 2 ms. As the focal length of one of the liquid lenses and the working distance of the laparoscope change, the captured images may defocus. By selecting the ROI in the viewed image, the contrast between the present ROI image and the previous ROI image is compared by Sobel operator detection and a peak search is conducted to find the best focusing position. Current control is then sent to the liquid lens to conduct quick focus. Auto-focusing of the laparoscope on the selected ROI can be achieved as the laparoscope moves or changes magnification at a speed of around 1.5s.

### B. Monocular Depth From Defocus Training Pipeline

To deal with inconsistent camera intrinsic due to magnification change, defocus cues are introduced as an alternative way to perceive depth. The depth from defocus (DFD) technique [27] provides better stability compared to feature-based matching methods [28]. Traditional depth from defocus requires at least 2–3 pictures from a focal stack to estimate depth. To correctly estimate depth from single monocular images, we model the focal stack by preprocessing blur on both synthetic and real laparoscopic images, and train our network with these preprocessed different focal lengths pictures.

The defocusing in laparoscopes is modeled on two abdomen datasets, one synthesized from Unity [29] and the other obtained from SERV-CT [30]. The first dataset provides synthetic laparoscopic images with different textures and with ground truth depths. This dataset is based on pseudonyms taken from the original IRCAD 3D CT liver dataset. The SERV-CT dataset is a stereo-endoscopic reconstruction validation dataset based on cone-beam CT. 1900 photos and their corresponding depth from the first dataset and another 30 real laparoscopic images from the SERV-CT dataset are archived.

We model the defocusing effect in laparoscopic surgery using a thin lens model. Assuming the object-space distance is $S_{1}$. The image-space focal distance $f_{1}$ equals $\frac {FS_{1}}{S_{1}-F}$, $D$ is the aperture diameter. The size of the circle of confusion (COC), denoted as $c(x)$, pertains to a 3D point positioned at the object distance $x$ and can be defined as:

\begin{equation*} c\left ({x }\right)=\alpha \cdot \frac {\left |{ x-S_{1} }\right |}{x}, ~\mathrm {where}~ \alpha =\frac {f_{1}}{s_{1}}D \tag{2}\end{equation*}

To model the defocusing effect in real laparoscopic surgeries, gaussian blur is manually conducted on the images mentioned above to synthesize the COC diameter. The object-space focal distance $S_{1}$ is set in most laparoscopic surgeries at around 100-150mm, and image-space focal distance $f_{1}$is set ranging from 25 mm to 86 mm. The aperture diameter $D$ is 10 mm.

To simulate defocus effect in laparoscopic images, we convolve the image with Gaussian blur function based on the different depth values of each pixel, and obtain a set of defocused images formed at different focal lengths. The depth values of the image come from the ground truth depth image and the working distance we defined. A stack of laparoscopic focus images includes the original unfocused data and four subsequent simulated defocused images calculated at randomly chosen $f_{1}$ and $S_{1}$. For each pixel, the corresponding $c\left ({x}\right)$ is calculated according to the given $f_{1}$ and $S_{1}$ through formula (2). The Gaussian function used for filtering is calculated based on $c(x)$, where the kernel standard deviation $\sigma (x)=c(x)/k$. $k$ is a constant that is related to lens parameters such as aperture diameter. Here, we empirically set $k=2$. In digital image processing, the kernel size of Gaussian blur is always odd, and the kernel size of Gaussian blur is calculated as:

\begin{equation*} r=round\left({max\left({0,\frac {\sigma \left ({x }\right)-0.8}{0.3}+1}\right)}\right)\times 2+1 \tag{3}\end{equation*}

We divide the image into four discrete layers based on depth range, each layer using a Gaussian function of the same kernel size, and then merge them to obtain the image with simulated defocus. By computing four different sets of $f_{1}$ and $S_{1}$, preprocessed laparoscopic focal stack images are obtained and used to train the proposed network below.

### C. Depth Estimation Network

**FIGURE 3.**
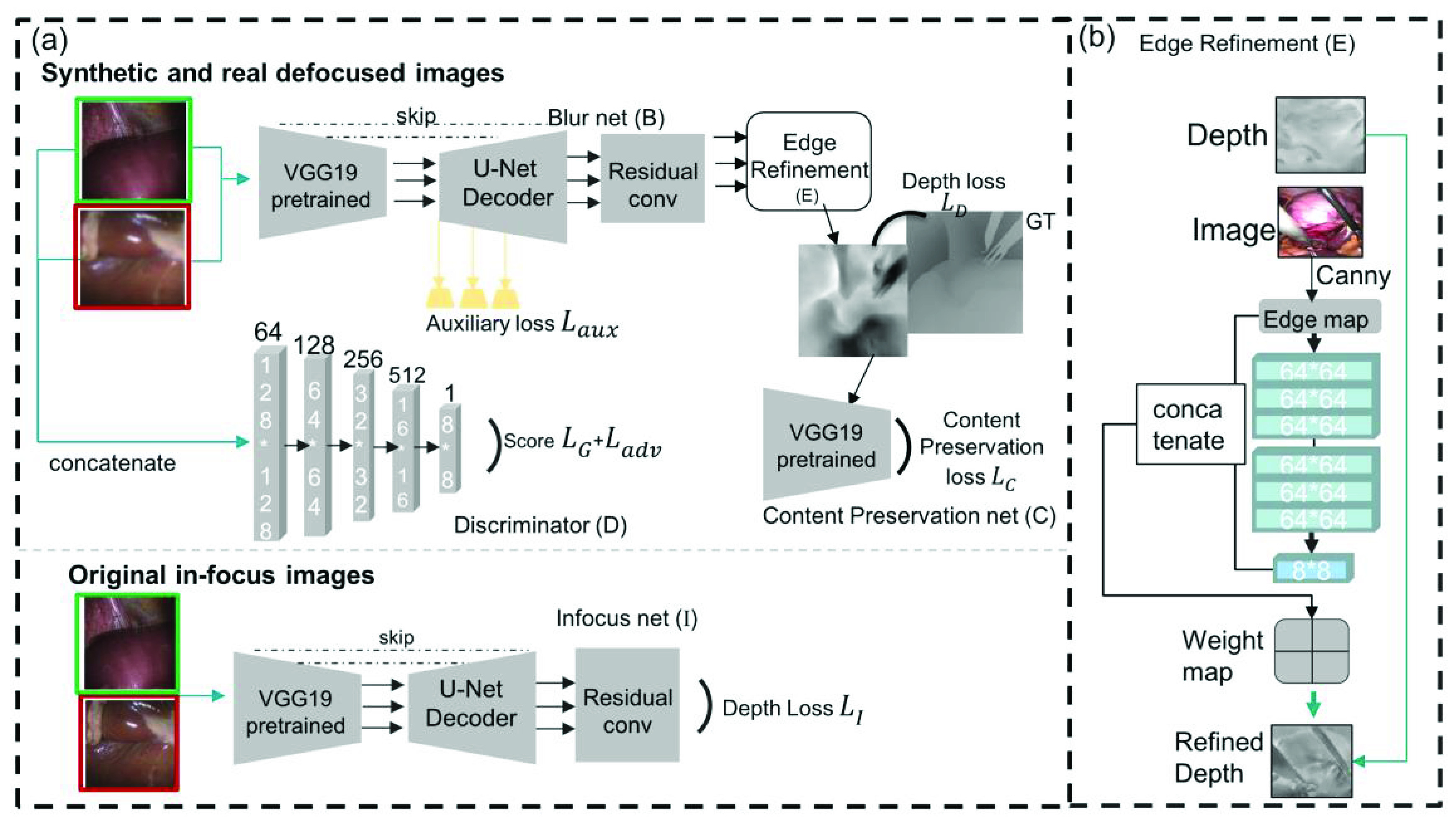
Proposed architecture of Monocular Depth from Focusing Net (MDFNet). (a) The upper part shows the network pipeline for synthetic and real defocused images, the bottom part shows the network pipeline for real in-focus image training. The green and red boxes show the corresponding in-focus and defocus image pairs. (b) The edge refinement part of MDFNet.

The proposed depth perception network MDFNet, shown in Fig. 3 (a), consists of two subnetworks: the depth-from-infocus-images net **I** and the depth from defocus net. The defocus network has three parts: generative adversarial network, including the depth-from-blur-images net **B** as the generator, and the discriminator **D**, the edge refinement module **E**, and the content preservation module **C**. Details of each submodule are as follows:

The depth-from-blur-images net **B** is based on a pretrained VGG-19 encoder and U-Net decoder with residual convolution. Short skip connections are used to refine domain-adapted features. Scale-wise auxiliary loss L_aux_ is used in our network at each up-sampling layer to guide multi-scale prediction of the depth map.

Network **B** acts as a generator and our discriminator **D** is based on 4 convolution layers with leaky rectified-linear-unit (ReLU) activation. Due to the size gap between real and synthetic laparoscopic images, a generative adversarial network is essential. The discriminator and generator work in a competitive way to help train the network to adapt from synthetic images to real abdomen images.

After the convolution blocks of network **B**, an iterative edge refinement module **E**, similar to the edge refinement of [31], is connected, as shown in Fig. 3 (b). The edge graph of the original image is computed using the Canny function and convolved by double-three convolution layers. The edge map detected by the Canny function is convolved as the edges of speculation are also detected in the initial edge map. Convolution layers help learn deeper edge features, reducing specular effect and fitting the convolved size of the initially estimated depth. The final convolution result is superimposed with the initial edge graph, outputting an edge weight graph. The weight is then iterated on the depth region by region, which helps refine the edges of the estimated depth output.

A content preservation module **C** composed of pretrained VGG-19 decoder is followed. Network **C** adds content consistency to the predicted depth and is trained using the similar loss of [32].

The depth-from-infocus-images net **I** shares the same structure as network **B**, and both these two networks are trained using the mean square error (MSE) loss, specifically the loss of the in-focus image depth estimation as $L_{\mathrm {I}}$ and the loss of the defocused image depth estimation after edge refinement module as L_D_.

The network is first pre-trained on the KITTI dataset and then fine-tuned using the previously mentioned preprocessed datasets. Both preprocessed defocused real and synthetic images are inputted into the generator **B** and the discriminator **D**. For data augmentation, Gaussian noise is randomly added. The corresponding in-focus laparoscopic images are inputted into the depth-from-infocus-images net **I**. For each depth map, four differently defocused image and one in-focus image are inputted, which act as a focal stack and provide different features. The in-focus and defocus image of the same laparoscopic scene is trained by network **I** and **B** correspondingly.

The depth-from-infocus-images net **I** is trained with loss $L_{\mathrm {I}}$, the defocus network with loss L_D_, the U-Net encoder with auxiliary loss L_aux_, the content preservation network with loss L_C_, and the generative adversarial network with loss L_G_ and L_adv_, as labeled in Fig. 3. The networks are trained in two paths, namely the in-focus laparoscopic depth estimation path and the depth from defocus cues path. After several experiments, the two routes each accounting for 0.5 give the best training result. The training goal is to minimize the sum of the aforementioned losses:

\begin{align*} L_{all}&=0.5\ast L_{I}+0.1\ast L_{D}+0.1\ast L_{aux} \\ &\quad +\,0.1\ast L_{G}+0.1\ast L_{advz}+0.1\ast L_{C} \tag{4}\end{align*}

## III. Results

### A. Prototype and Capsulation

**FIGURE 4.**
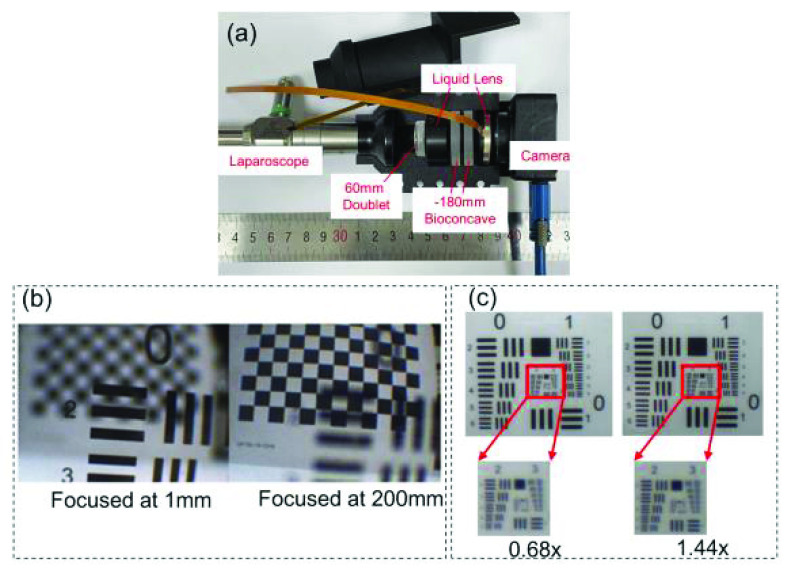
(a) Overall system capsulation view of the lenses (b) The focusing ability at different working distances (c) Comparison images of USAF 1951 resolution target taken at 0.68x and 1.44x magnification rate.

According to the optical system described above, a plastic shell is designed for assembling the lenses. The plastic shell is a symmetric structure and has holes on top of the lenses for the flex cable of the tunable lens, as well as holes along the sides of both the upper and lower part to immobilize the structure. The eyepiece of the laparoscope and the C-mount of the CCD are also considered in the model. It is then 3D printed and matt black coated, as shown in Fig. 4(a). The overall length of the system is 94mm, with a weight of 147g.

### B. Magnification Rate

According to the optical structure mentioned above, a magnification rate of 0.68x-1.44x can be achieved.

The focusing ability at different working distances is tested, as shown in Fig. 4(b). The image quality at different magnifications is also tested, as shown in Fig. 4(c). References [25] and [26] stated that multi-zoom optical structures for laparoscopes suffer from poor light and distorted colors. The results show that our proposed system can provide quick auto-focus with working distance between 1mm and 200mm, and show better stability to light and color constancy, achieving at least 5.04lp/mm for a 1.44x magnification rate, and 4.00lp/mm for a 0.68x magnification rate at 100mm working distance, according to Rayleigh criterion. Therefore, at a working distance of 100mm, the spatial resolution of the system is $0.27~\mu \text{m}$/pixel and $0.34~\mu \text{m}$/pixel at 1.44x and 0.68x magnifications, respectively.

### C. Depth Estimation

The MDFNet network is trained using Nvidia 1080ti and under 20 epochs, with a batch size of 1 and with an initial learning rate of 0.0001, decaying by 0.8 every 5 epochs. During the evaluation, the output depth maps $D_{out}$ and ground truth $D_{\mathrm {gt}}$ are compared under uint8 data type.

**FIGURE 5.**
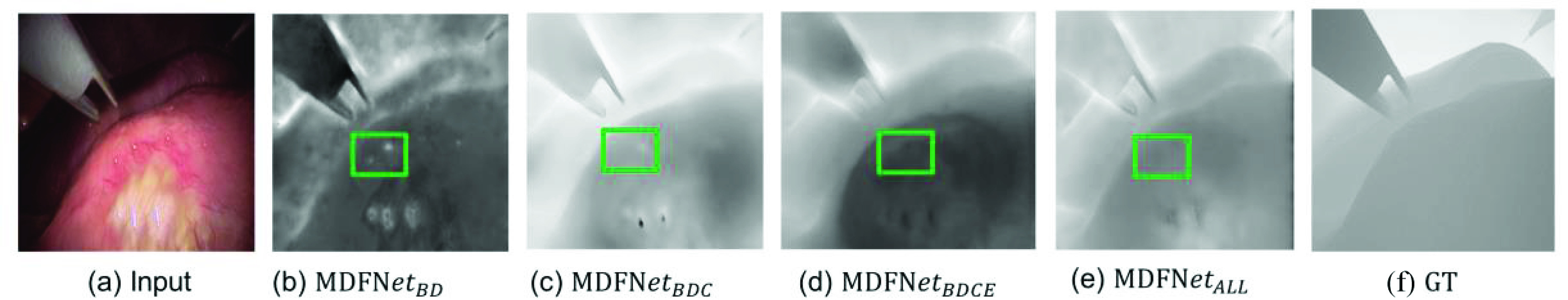
Depth outputs generated with different components of our network. The green boxes show that the added edge refinement module helps reduce specular issues. (a) is the original laparoscopic image input. (b) is the proposed network trained only with the depth-from-blur-images net B and discriminator network D, which means only defocused images are inputted. (c) is the proposed network trained with the depth-from-blur-images net B, the discriminator network D, and the content preservation network C. (d) is the proposed network trained with the depth-from-blur-images net B, the discriminator network D, the content preservation network C, and the edge refinement E. (e) is the network trained with both defocus images and in-focus images, and with all the modules described. (f) is the ground truth depth of the inputted laparoscopic image.

The effects of the different components of our network is shown in Fig. 5. As is shown, the depth-from-blur-images net **B** and discriminator network **D** learn the basic contours of the image but suffer from artifacts. The added content preservation network **C** reduces the artifacts and smooths the output but is sensitive to light reflection. Edge refinement **E** further differs the depth levels and lowers the influence of specular highlight, shown in green in Fig. 5. Adding depth-from-infocus-images net **I** means that the network is trained jointly on both defocus images and in-focus images, as shown in Fig. 5(e), provides a smoother depth output.

**FIGURE 6.**
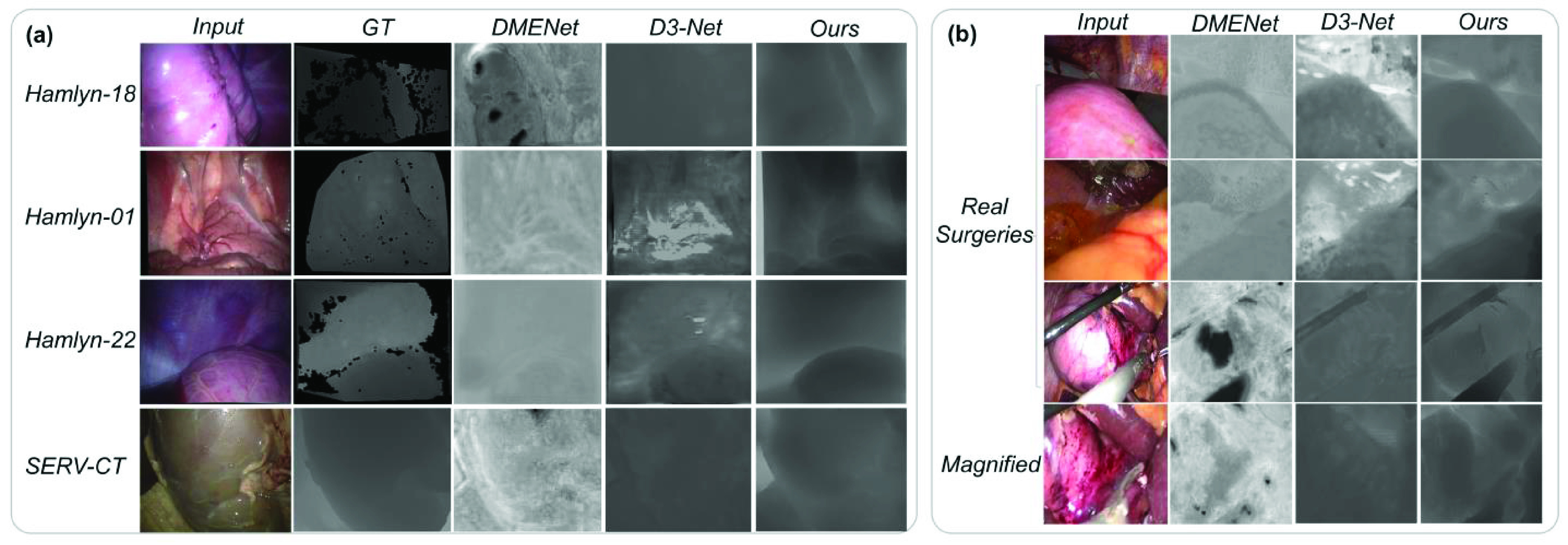
(a) Depth outputs of DMENet [27], D3-Net [29] and our proposed MDFNet on the Hamlyn dataset no.18, no.1, no.22, SERV-CT dataset, (b)real defocused and in-focus clinical surgeries images provided from Changgung Hospital real defocused and in-focus clinical surgeries images provided from Changgung Hospital using a Storz laparoscope (patients suffering from hepatolithiasis which need minimally invasive hepatectomy), with the last row a magnified view of the clinical scene, used to show our depth estimation of magnified scenes help improve the depth resolution.

As this is the first paper conducting multi-magnification and monocular depth estimation based on deep learning defocus cues in laparoscopic scenes, two depth from defocus networks proposed in the natural image field, namely DMENet [32] and D3-Net [33] are used as comparison. These two networks are trained on the same training data and tested on in-focus datasets, namely the Hamlyn dataset [34] of the abdomen images and the remaining SERV-CT dataset, shown in Fig. 6(a). The networks are also tested on unlabeled clinical laparoscopic defocused images provided by Beijing Changgung Hospital, as shown in Fig. 6(b). The last row in Fig. 6(b) shows the depth output of the magnified scenes of the image above. The two depth outputs show different depth details, which prove our method’s ability to improve resolution by estimating depth on different magnifications.

Results show that DMENet suffers greatly from specularity and fails to estimate depth in laparoscopic scenes. D3-Net performs better on specularity but still fails to give accurate depth and suffer most from outliers. As both the Hamlyn and SERV-CT test images are in-focus, the two networks do not suit small depth range situations like the abdomen and fail to estimate depth on in-focus images. On the contrary, MDFNet fits laparoscopic scenes better, and can estimate depth monocularly from images of different focal lengths, namely defocused images and in-focus images.

The performance evaluation of MDFNet compared with DMENet and D3-Net is shown in Table 1. The Hamlyn dataset provides stereo images and camera calibration intrinsic. The ground truth depth is then calculated by EndoDepth [35]. The results are compared under uint8 format. While for RMSE, as uint8 ranges from 0-255, the two depth images are normalized by dividing each value by 256 before comparison. The depth outputs show that the original DMENet and the D3-Net trained on laparoscopic data do not fit small depth range situations and both suffer from outliers. Our depth network is shown to also be able to estimate depth monocularly from in-focus images. However, as the ground truth depth is calculated according to the camera calibration intrinsic, the GT depth is a relative value and there may also be error caused during the GT calculation. We therefore further conduct system validation on ex-vivo models, and use 3D scanners for the real GT output.

**TABLE 1:** Evaluation Performance of Depth Estimation on Hamlyn Dataset

	PSNR	MAE(mm)	RMSE(Normalized)	Abs Rel(mm)	SSIM	$\delta < 1.25$	$\delta < 1.25^{2}$
DMENet [32]	56.019	11.535	0.460	0.223	0.650	0.804	0.968
D3-Net [33]	53.364	69.875	0.493	0.494	0.696	0.241	0.801
Ours	55.420	4.047	0.446	0.205	0.722	0.837	0.982

### D. System Validation

The overall performance of our system is validated in both qualitative and quantitative ways. The light source used in all experiments are the cold light source LED DELON 300 with a luminous power of 180W and a color temperature of 6500K.

**FIGURE 7.**
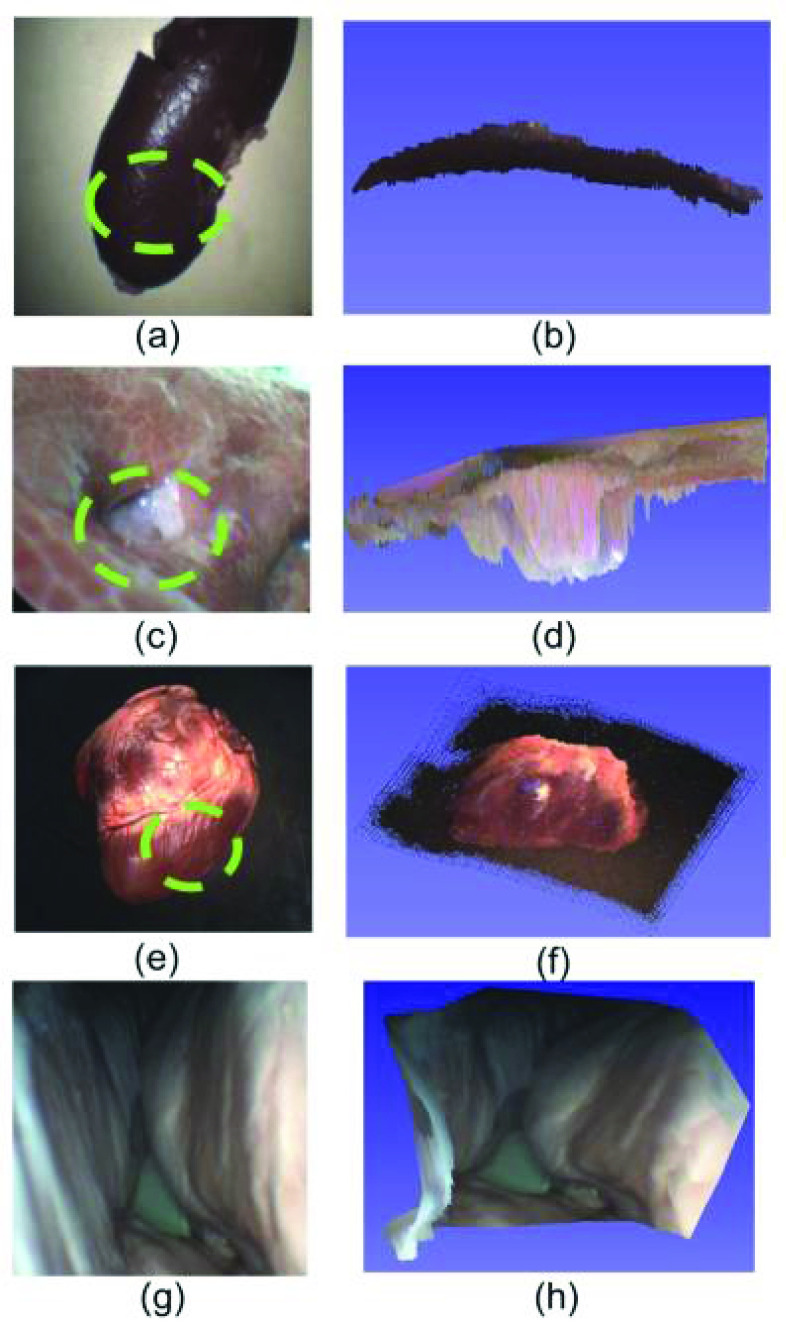
Ex-vivo experiment results. (a) Photo of a porcine kidney, the measured area is circled by the dashed circle (magnification ratio 0.68x, working distance 100 mm). (b) The reconstructed surface of the porcine kidney. (c) Photo of porcine liver with a hole (magnification ratio 0.68x, working distance 30 mm). (d) The reconstructed surface of the porcine liver with hole. (e) Photo of porcine heart (magnification ratio 0.68x, working distance 100 mm). (f) The reconstructed surface of porcine heart. (g) Photo of porcine lung (magnification ratio 0.68x, working distance 2 mm). (h) The reconstructed depth range of (g).

Ex-vivo experiments are conducted on a porcine kidney, a porcine liver, a porcine heart, and a porcine lung, as shown in Fig. 7(a), (c), (e), and (g). The reconstructed surfaces are shown in Fig. 7(b), (d), (f), and (h). The reconstructed surface curve agrees with the morphology of the organs. In Fig. 7(h), our system proves feasibility in near observation situations.

**FIGURE 8.**
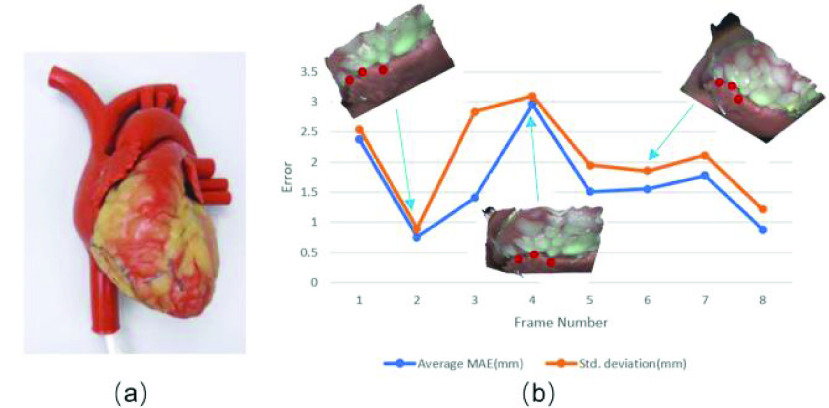
(a) Picture of the heart model, captured by the proposed system with magnification ratio of 0.68x and working distance of 150mm. (b) Reconstruction errors of three points tracked on a clock wisely moving rigid heart model. The inserted figures are the corresponding reconstructed surfaces at three instants, with the three measured points in red.

To further validate the robustness of dynamic scenes, a porcine heart model is reconstructed, whose ground truth point cloud is obtained via the Artec Eva scanner. The heart model is moved clock wisely, and the 3D reconstruction is conducted in real-time with a speed of 6fps. During the process, three points on the model are measured and the depths are compared with the ground truth, with the MAE results and the standard deviation values shown in Fig. 8.

**FIGURE 9.**
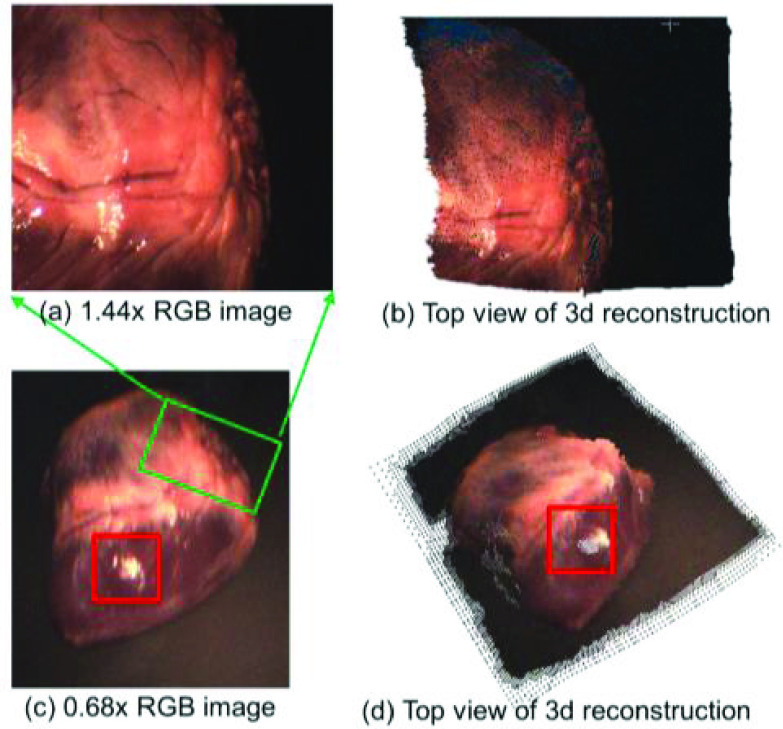
3D reconstruction of our proposed system on a porcine heart (working distance 100 mm). (a) shows the image under 1.44x magnification; (b) shows the 3D reconstruction of (a); (c) shows the captured RGB image under 0.68x magnification, and (d) shows the 3D reconstruction of (c). The red box in (c) shows that the proposed method still fails in strong speculation situations. The green box shows the corresponding magnified part of the porcine heart on the low magnification image.

Images of an ex-vivo porcine heart are then captured, and 3D reconstruction is conducted according to the depth estimation. The result further confirmed the feasibility of the proposed system. The Artec Eva scanner is again used to capture the 3D point cloud of the pig heart as ground truth. Fig. 9(a) shows the captured RGB image by our proposed optical structure at a high magnification rate, (b) shows the 3D reconstruction of the magnified view, and (c) shows the captured image at a low magnification rate, with (d) the corresponding monocularly 3D reconstructed porcine heart of (c). The red box in Fig. 9(c) shows that the proposed method still fails in strong speculation situations, which needs to be further improved. The green box in (c) shows the corresponding part of the high magnification view on the low magnification view. The depth estimation is 0.17s per image, tested on Nvidia RTX 3080.

**FIGURE 10.**
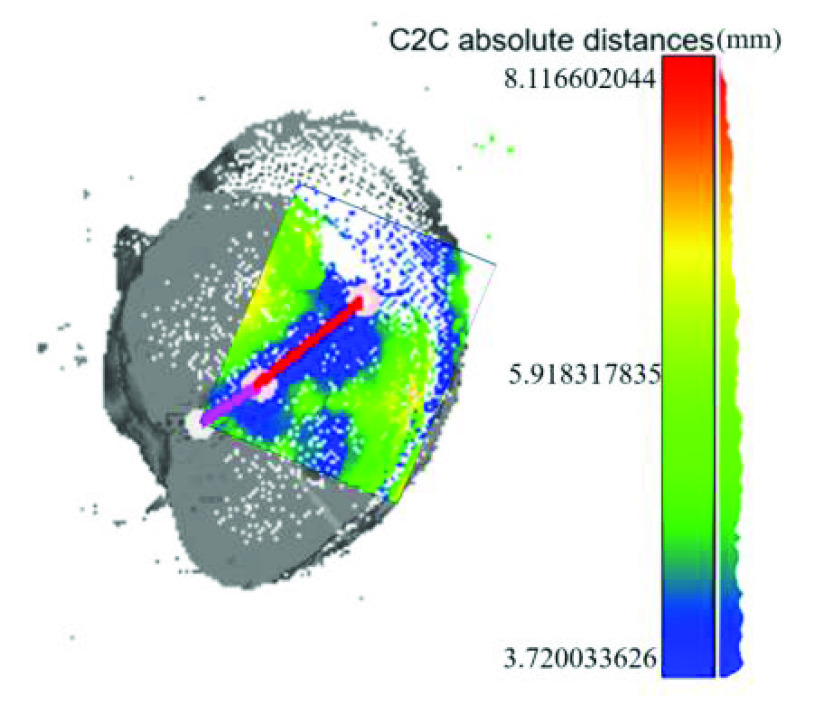
Distance heat map between 3D reconstruction alignments of the porcine heart (colored), with two parts of a blood vessel (purple and red lines) measured (magnification ratio 0.68x, working distance 100 mm).

We again normalized and aligned our point cloud of the porcine heart captured under high magnification rate with the ground truth in CloudCompare, shown in Fig. 10. The 3D reconstruction of the porcine heart in Fig. 10 is based on the monocularly captured right view in Fig. 9(a), corresponding to the upper right part of the porcine heart, which is colored in Fig. 10. The distance heat map between the 3D reconstruction of the porcine heart by our system and the 3D scanner is shown. The depth of the whole porcine heart varies from 0mm to 62.07mm, while the colored part varies from 0mm to 41.50mm. We measured two parts of the blood vessel on the porcine heart, drawn in red and purple on the heat map. The length of the vessels is 44.198mm and 37.108mm measured by our system, while the ground truth length is 40.03mm and 32.06mm correspondingly, given by the 3D scanner Artec Eva. The blood vessel length estimation average error of our system is 4.61mm. The overall registration error is shown in the color bar, with the blood vessel area averaging at around 4.39mm. The above results show that our system can give a reference length estimation of anatomical morphological features like blood vessels, which can help surgeons get better length and size concept of the abdomen during laparoscope surgeries. Different viewing angles and magnification rates should be blended to achieve a higher resolution and smoother overall reconstruction in the future.

We also conducted consecutive depth estimation, our system can estimate depth on 7 consecutive frames captured under different focal lengths, meaning the blurriness and the magnification rates of the image are different, so the image resolution is also different. The average depth error is 0.78mm, with standard deviation 0.22mm, proving that our system can provide stable depth estimation under different imaging qualities, which is the advantage of our proposed method.

## IV. Discussion

While most studies in the literature focus only on designing bulky structures to provide a field of view change or only on combining depth estimation with semantic segmentation, camera pose, or scene flow estimation, we proposed integrating a multi-magnification system with real- time 3D reconstruction ability. The proposed system was evaluated both on its imaging ability and depth estimating ability.

The experimental results confirm that our system promises to address limited magnification, blurred near imaging and lack of depth perception. As compared to another monocular laparoscopic system which enlarges FOV and provide depth perception [6], our system shows slightly higher reconstruction accuracy (5.31mm vs 4.608mm). Compared to a stereo system composing of a stereo laparoscope with 6mm baseline distance and Monodepth2 [36] depth perception method, our system shows smoother and higher depth results (7.69mm vs 6.35mm).

Compared to other studies providing near observation [22] and multi-magnifications [23], we substituted the need for such complex and bulky designs with a portable off-the-shelf solution that can fit conventional laparoscopes. As camera intrinsic is inconsistent, we introduce using defocusing cues as an alternative way for real-time monocular depth estimation and 3D reconstruction. Compared to other state-of-the-art depth estimation methods in laparoscopy, [15] and [16] require consistent camera intrinsic, which fails in multi-magnification situations. Our depth estimation results are compatible in smoothness, speed, and accuracy, and we can work well with images of different focal lengths. Compared with stereo laparoscopes like Olympus, our system shows better depth estimation accuracy in specularity and smooth organ surfaces, the 3D reconstruction error of our system is also slightly better than stereo laparoscopes. Compared with Rubina series of Karl Storz, our system does not require 3D glasses and can further provides magnification changes through optical zoom. Therefore, the proposed integrated system can achieve multi-magnification, autofocus, near observation, and real-time 3D reconstruction all in one.

The above advantages state our system can provide better reference during laparoscopic surgeries. Our system is more specifically targeted at global and magnified imaging as well as depth estimation of incisions, blood vessels, fluorescence and other anatomical morphological features. We provide a monocular depth estimation system which help surgeons get better length and size concept of the abdomen during laparoscope surgeries. With the ability to change views and the given reference depth as well as 3D reconstruction, surgeons can conduct diagnosis of lesions more efficiently. The system also reduces depth uncertainty caused by limitation of optical imaging resolution.

The influence of depth resolution on clinical detection and diagnostic decision-making is pivotal in evaluating the practical implications of our proposed system. A higher depth resolution can enhance the precision of lesion localization, tissue characterization, and overall diagnostic accuracy. Conversely, limited depth resolution can introduce challenges in accurately assessing the spatial relationships between anatomical features, particularly in scenarios where precise depth cues are integral to distinguishing between healthy and pathological tissues. The integration of our proposed multi-magnification deep learning depth estimation into clinical settings introduces an innovative dimension to these considerations. Addressing the challenges posed by depth limitations can contribute to more accurate and informed clinical decision-making. Further research and validation, including the assessment of sensitivity and specificity, will be pivotal in establishing the clinical benefits and applicability of our proposed approach within the dynamic landscape of surgical interventions.

However, our system still needs to increase the magnification range and may fail in extreme situations such as smoke occlusion and blood flow with strong specularity. We further discuss the future focus of our system as follows.

To improve the depth accuracy of our deep learning-based depth estimation algorithm, we have addressed the limitations posed by optical resolution of the laparoscopic images. Our solution was to implement an optical zoom feature in our system’s design, which covers a range of 0.68x to 1.44x. This zoom functionality is critical in enhancing the optical resolution as the magnification of the laparoscopy increases. However, the optical design is constrained by factors such as volume and weight of the optical structure of multiple lenses, and the maximum magnification range achievable is 1.44x. This range may not be sufficient for imaging smaller lesions, such as tumors, in clinical applications. To expand the potential clinical use cases of our system, we need to consider further increasing the optical zoom range.

Artifacts and noises are the drawbacks of estimating depth from defocusing cues. As our 3D reconstruction is conducted monocularly, in the future, we intend to conduct 3D reconstruction on multi consecutive frames under different viewing angles. As the resolution under different magnification rates is different, we also propose to conduct 3D reconstruction based on multi-consecutive frames in the future. During the conducted experiments, a 3D reconstruction model captured under low magnification rate has 3527781 points, whereas a 3D reconstruction model captured under high magnification rate has 4496022 points, therefore blending the two images before 3D reconstruction can improve accuracy and density, which shall be our future focus.

As proposed in [37], different focus rate images are captured as the laparoscope moves towards the neoplasia for size estimation. In our system, after the depth estimated from MDFNet, we can also obtain the real-time focal length of the system and further conduct neoplasm size measurements.

As using the robot arm for laparoscopic control is proposed in [38], our proposed liquid lens optical structure can autofocus on the selected ROI, which can further be coordinated with the robot arm and accomplish an integrated diagnosis and treatment system.

In laparoscopic procedures, minimizing surgeon overhead and optimizing workflow efficiency are crucial considerations. The proposed system requests manually selecting the ROI for autofocus adjustment each time the laparoscope shifts or the image is magnified can potentially burden the surgeon and interrupt the surgical flow. In future studies we will integrate automatic ROI tracking into the proposed system. This advancement seeks to empower the system to autonomously detect and track the pertinent ROI as the laparoscope moves or the field of view is adjusted. By doing so, the need for repeated manual ROI selections is obviated, streamlining the autofocus process and allowing the surgeon to maintain their focus on the surgical task at hand.

## V. Conclusion

Modern laparoscopic surgeries pose several specific challenges such as fixed magnification rates, physical focusing, and lack of depth perception. We have proposed a laparoscopic system including an integrative optical structure for conventional laparoscopes using two liquid lenses and three optical lenses to obtain multi-magnification rates, auto-focusing, and near observations, as well as a depth network for real-time monocular depth estimation and 3D reconstruction of photos under different focal lengths. The proposed optical structure has been shown to exceed current liquid lens-based optical structures in both light stability and weight. The real-time deep-learning approach can estimate depth monocularly from laparoscopic scenes without restriction to camera intrinsic. The system can perceive depth monocularly in laparoscopic scenes at a rate of 6 fps, and less than 6 mm in error. Results also show that jointly training the network with in-focus and defocused image pairs helps perceive depth in a smoother and edge-aware way and provide a way to cope with texture-less environments. This method provides an alternative to state-of-the-art laparoscopic 3D reconstruction techniques and proves its feasibility. The system is targeted at providing multi-magnification view of anatomical morphological features during laparoscopy, and give surgeons depth and size reference to aid their diagnosis.

The proposed laparoscopic system offers the advantages of low cost, lightweight, multi-magnifications, near observations, and real-time depth estimation. Therefore, the proposed system can be regarded as a favorable system prototype for integrated imaging and diagnosis in laparoscopic surgeries.
